# Electromechanical Assessment and Induced Temperature Measurement of Carbon Fiber Tows under Tensile Condition

**DOI:** 10.3390/ma13194234

**Published:** 2020-09-23

**Authors:** Samir Mekid, Hammam Daraghma, Salem Bashmal

**Affiliations:** Mechanical Engineering Department, King Fahd University of Petroleum and Minerals, Dhahran 31261, Saudi Arabia; g201401700@kfupm.edu.sa (H.D.); bashmal@kfupm.edu.sa (S.B.)

**Keywords:** carbon fiber, characterization, tensile test, smart material

## Abstract

The paper presents an investigation and analysis of the electromechanical and thermal characteristics of the carbon fiber alone as single tow and embedded in host materials such as polymer e.g., acrylonitrile butadiene styrene (ABS) using 3D printing. While carbon fibers can partially reinforce the structure, they can act as sensors to monitor the structural health of the host material. The piezo-resistive behavior was examined without any pretreatment of the carbon fiber under tensile test in both cases. Special focus on the filaments clamping types and their effects was observed. An auxetic behavior was exhibited; otherwise, the free part shows elastic and yielding ranges with break point at high resistance. An induced temperature of the carbon fiber was measured during the tensile test to show low variation. The carbon fiber can provide strength contribution to the host material depending on the percentage of filling the material in 3D printing. The relative variation of the electrical resistance increases by 400% while embedded in the host material, but decreases as the tows filament density increases from 1 to 12 K.

## 1. Introduction

Ensuring the operational safety and long-term stability of structures are essential requirements in designing and improvement of new materials. Integrating a structure with a sensory system that mimics the nerve system in its ability to sense, locate, and heal damages is an ultimate goal that researchers are taking steps toward its realization [1]. This is immediately followed by adding embeddable low power actuators for actions [2]. As an essential step, many researchers attempted to enhance the health monitoring capability by embedding sensors within the structure. Several candidate smart materials, such as optical fibers, carbon fibers, piezoelectric, electromagnetic and others, are examined and show promising performance in structural health monitoring. However, researchers are faced with many challenges related to their sensitivity, reliability, durability, integrity, etc. Embedded sensors should not compromise the strength of the structure, provide an accurate and comprehensive inspection, and require minimum external intervention or maintenance [1].

Superior mechanical properties of carbon fibers make them of particular importance in structural reinforcement. The most prominent characteristic of the carbon fibers (CFs), in spite of their low density, are their relatively high strength and stiffness compared with steel. In addition, the aspect size ratio boosts their ability to be reinforced within different polymer-based materials. Such reinforcement was utilized to enhance the strength properties of the hosting structures in various applications. For instance, aerospace applications are widely using CF-reinforced composite polymers [2,3,4].

Moreover, the carbon fibers possess excellent sensing ability due to their linear piezo-resistive behavior. In recent years, researchers realized that variation in the fibers’ electrical properties can be used as a mean to quantify mechanical strain [5]. The piezo-resistive behavior refers to the ability of sensing the deformation behavior i.e., strain, by measuring the resistance changes. The piezo-resistivity of CFs could become a promising technique for structural health monitoring to those structures containing the CFs, where the strain change of the material can be monitored by a resistance change [6,7,8]. In this regard, the CFs can act either as a sensing sensor or damage sensor by measuring the resistance variation under mechanical strain [9,10,11]. This resistance is defined by Equation (1):

(1) $$R=\frac{\rho \;L}{A}$$

where R is the electrical resistance (Ω), ρ is the resistivity or specific resistance (Ω.m), L and A are the length and the cross-sectional area of the fiber, respectively.

The relative resistance change δR/R can be described by Equation (2) [12,13]:

(2) $$\frac{\delta R}{R}=\frac{\delta \rho }{\rho }+\varepsilon \;\left(1+2v\right)$$

where δρ/ρ resistivity change, ε is the longitudinal strain defined as δL/L, and ν is the Poisson ratio. The gauge factor (k) is an essential indication describing the piezo resistivity behavior of the CFs under the longitudinal strain. The gauge factor (k) is defined by the ratio of the resistance variation to the strain applied δρ/ρ /δL/L.

Carbon fibers in the elastic region exhibit different tensile modulus behavior i.e., non-Hookean behavior [14,15,16]. The crystalline orientation, as well as the microstructure style, are standing behind the non-Hookean behavior of the CFs [17,18,19,20]. Under longitudinal tension, CFs are mainly characterized by low modulus and high modulus. 

**Figure 1:**
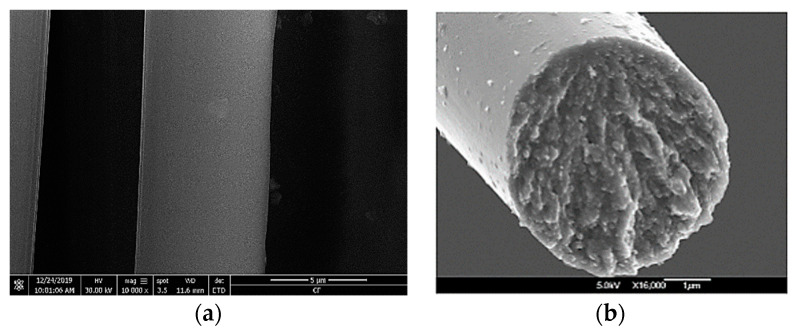
SEM micrographs of (**a**) our single fiber and (**b**) fiber tensile fracture surface at surface cross section from [24].

Characterizing the behavior of carbon fibers under tensile testing is crucial to understand the structural flaws that lead to fibers failure Figure 1. For example, Penev et al. [21] explored the mechanical behavior of carbon fibers using large-scale molecular dynamics simulation. It is found that cracks are initiated at the interface of the mis-oriented crystallite with the surrounding regions. Okuda et al. [22,23] attempted to understand the effect of nanostructure on the tensile strength of carbon fibers. 

According to the authors, large flaws in the carbon fibers can significantly deteriorate the fiber strength. It is also suggested that, in absence of large flaws, the disordered region is most likely contributing to the tensile strength more than the crystallites. Few papers where cited that discussed the tensile failure phenomena in carbon fibers from micro- and nano-structural points of view. Possible causes of failure in carbon fibers are crystallite shear failure, failure of wrinkled crystallites, rupture of the basal planes within misaligned crystallites, and yielding of the disordered structure [23]. Using nano-indentation techniques allows the extraction of fracture toughness and residual stress of the carbon fibers at the nanoscale as described in [25].

The resistivity change during tensile condition contributes to the piezo-resistivity behavior of CFs and the gauge factor’s value reflects the resistivity behavior i.e., negative or positive behavior. With the absence of resistivity change effect, and for the typical value for Poisson’s ratio of 0.28, the limit value of the gauge factor is 1.56 [26]. The increasing or decreasing on the latter value will show the piezo-resistivity behavior. In general, increasing the applied strain leads to the variation of crystalline structure, consequently, the gauge factor is affected due to the resistance variation. Blazewicz et al. reported in [27] that relatively high modulus CFs exhibits negative piezo-resistivity i.e., −8.9. By contrast, the low modulus CFs shows relatively a higher gauge factor of 2.8 i.e., positive piezo-resistivity. Different previous works stated that piezo-resistivity behavior is either negative or positive, with a diversity of gauge factors’ values [28,29,30,31]. Embedding smart materials into structures to utilize their properties for actuation and sensing applications is an emerging topic that recently attracted the intension of researchers. It falls within the scope of developing a nervous system in the host material that can detect, diagnose, and quantify damages [1]. Saheb and Mekid presented an intensive revision of smart fibers and potential embedding processes with focus on optical fibers and metallic host materials [32]. Extensive numerical and experimental works were carried out to understand the parameters that controlled embedding of fibers using ultrasonic consolidation [33,34]. While methods like ultrasonic consolidation [33] and laser-based layer manufacturing are successful in sub-surface embedment, placement of fibers inside materials while ensuring their integrity and functionality remains as challenging task for researchers [32]. Mekid et al. evaluated the performance and integrity of embedded fiber optics under high pressure and temperature conditions [35]. Fibers were manually placed in ABS printed samples with different shapes and patterns and characterized under tensile, bending, and heat loads. For carbon fibers, earlier researchers evaluated the self-sensing properties in carbon fibers polymer composites to dynamic strains [36]. Chung provides a comprehensive review on structure property relationships of the continuous carbon fiber polymer-matrix composite [37]. The literature is rich with studies concerned with the self-sensing ability of carbon fiber composites and measurement techniques. 

Moreover, there are attempts to embed carbon fibers on other non-polymeric-based materials for health monitoring applications. Horoschenkoff embedded carbon fibers on the surface of glass fiber reinforced polymer specimens and pressure vessels to monitor crack density [10,38]. Based on the integral strain measurement property of carbon fiber, Matzies et al. proposed to use a network arrangement of the sensors, where a mesh is created on an acrylic glass plate to measure a two-dimensional strain field [11]. Recently, Goldfeld et al. investigated the feasibility of intelligent textile-reinforced concrete embedded with carbon fibers to add strength and sense damage at the same time [39]. Cracks were detected and it was shown that electrical readings can detect wetting of cracked elements which can be used to monitor leakages. 

More recently, Xi and Chung reported that carbon fibers have piezoelectric behavior [26]. It was indicated that although the detected permittivity is small for energy harvesting applications, it is sufficient and reversible to be considered a self-sensing property. In a series of publications, carbon fibers were embedded in a 3D printed structure to examine the self-sensing ability due to different types of loadings and defects [6,40,41,42]. A network of carbon fibers was meshed into 3D printed thermoplastic material and were successfully used to identify damage location. 

In tensile testing of fiber tows, care must be taken to reduce slippage and inhomogeneous stress levels. While the engineering standards recommended the use of end tabs or adhesive at the end of the specimen, no explicit procedure was identified to specify the tab materials, grip pressure, or grip alignments. Sawada and Shindo emphasized the importance of the clamping method during tensile testing of fiber tows [43]. The use of tabs at both ends of the fibers allows the distribution of the load and reduces the chance specimen failure due to bending moments. One sign to indicates that failure occurs due to bending is when the fracture happens at the joint near the clamps. Following the standard in tensile testing of fibers, researchers used tabs of different materials and sizes in order to avoid failure that is not pertained to pure tension of the specimen. Horoschenskoff et al. illustrated that use of tabs reduces the undesired strain levels at the electrical connection and stabilize resistance measurements [44]. Wang and Chung showed that there is an initial decrease on the resistance of carbon fibers embedded in the epoxy when subject to tensile stretching [45]. This is due to the compressive residual stress initiated during embedment and the curing process. For resistance measurements, several techniques are used in the literature to detect variation in the resistance: silver paints [46], copper soldering [47], clips [6], wrapped wires [8,48]. For carbon fiber composites, the commonly accepted method to measure electrical resistance is lacking. One of the main issues is to ensure perfect contact and prevent slippage of electrodes during tensile testing without causing localized stresses. In the current study, sandwiched aluminum foils or copper soldering are used in addition to the electrode clips as discussed in the next section.

This paper will consider the characterization of the bare carbon fiber in its initial form without any pre-treatment e.g., adding resin, with a particular emphasis on the clamping and port attachment with its effect on the overall behavior. This study will be followed by the characterization of the same carbon fiber under the same condition of no pretreatment while embedded in a host material e.g., polymer. The characterization will include the mechanical, electrical, and thermal behavior under tensile test in normal and cyclic modes. The results will be discussed with respect to the literature review.

## 2. Experimental Setup and Sample Preparation

### 2.1. Experimental Setup and Material

The experimental setup is described in detail in Appendix A.

### 2.2. Sample Preparation

In this experiment, the typical challenges are to secure vertical straightness of the CF with perfect gripping of the two ends avoiding slippage, bending moment, and stress concentration. In addition, the CF needs the electrodes to be connected for the real-time measurement of electrical resistance during the test. Two flat plates of Perspex material have been used to hold the CF and aluminum foil in sandwich. This has proved to be efficient in securing complete electrical conductivity by keeping the CF smooth, straightened, and free of stress concentration after several initial tests.

**Figure 2:**
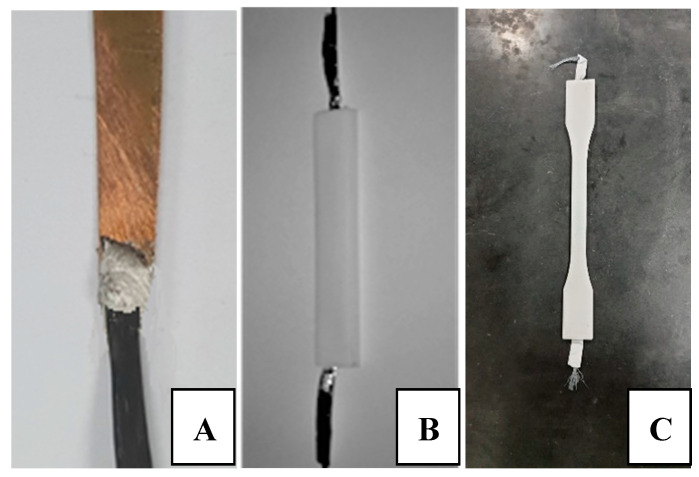
(**A**) Soldered port to copper, (**B**) ABS printed samples, and (**C**) printed to standard sample.

The second sample was built by embedding the CF inside ABS polymer using shapes shown in Figure 2B,C and overall dimensions of 20 × 10 × 140 mm. The ABS was melted at a temperature of 240 °C to be shaped as the printing progresses. The infill ratio used is 100% with speed of 60 mm/s. During the printing, the CF was inserted manually in the middle of the sample’s height with a certain tension. The ending parts had both aluminum added to secure conductivity.

### 2.3. Grips Analysis

**Figure 3:**
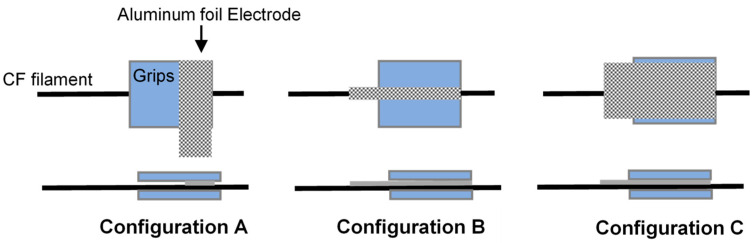
Port configurations.

The gripping system significantly contributes to the resistance measurements during the tests. Connections of the CF with copper ports using C-Solder from the market helped to form strong bonds that are thermally and electrically conductive, see Figure 2A. However, during the tests, the soldering breaks before reaching high-tension forces. As an alternative gripping technique, the CF filament was added to two Perspex small plates with the aluminum foil as the electrode for conduction. Three configurations were tested as shown in Figure 3 within two Perspex plates but different aluminum surface distribution. Configuration A with a lateral partial aluminum on the CF, configuration B with a longitudinal aluminum on the CF only, and configuration C aluminum covering all the surface of the Perspex. Some variation is observed in the measured resistance based on the configuration used and will be discussed.

## 3. Experimental Test

### 3.1. Preliminary Test

As a preliminary test, the three different configurations shown in Figure 3 were tested using 1 K CF filaments under longitudinal tension to investigate the effect of the gripping method on the electric resistance measurements. These three configurations have exhibited mainly three different regions during the test with respect to strain; initial region with increased load showing small decreasing of the resistance called here “preloaded or gripping region”. This region is affected by the through-thickness resistance phenomena i.e., the contacts between the fibers due to nascent squeezing of the fiber between the grips also explained in Ref. [5]. As the load increases, the resistance is noticeably increasing by relatively high slope; this region is called “free CF length”. This increasing happens due to the actual extension in the gauge length and consequent reduction in the cross-section as seen in Equation (1).

**Figure 4:**
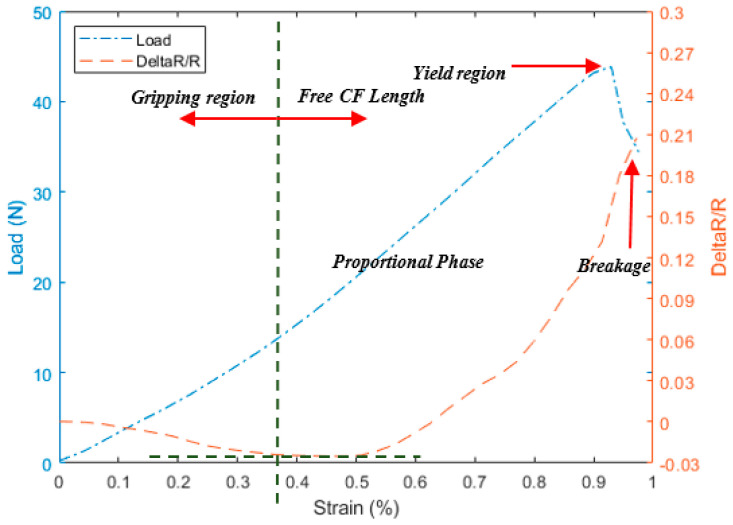
The tensile test for 1 K and the corresponding resistance variation measurements for configuration A.

**Figure 5:**
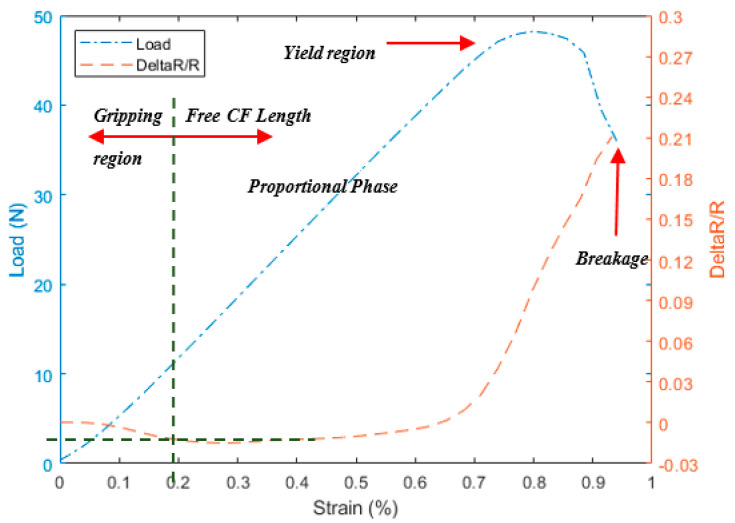
The tensile test for 1 K and the corresponding resistance variation measurements for configuration B.

**Figure 6:**
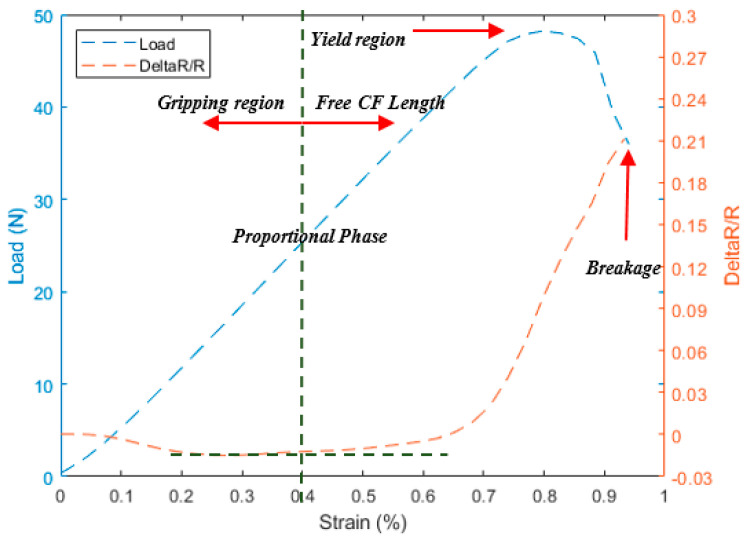
The tensile test for 1 K and the corresponding resistance variation measurements for configuration C.

**Figure 7:**
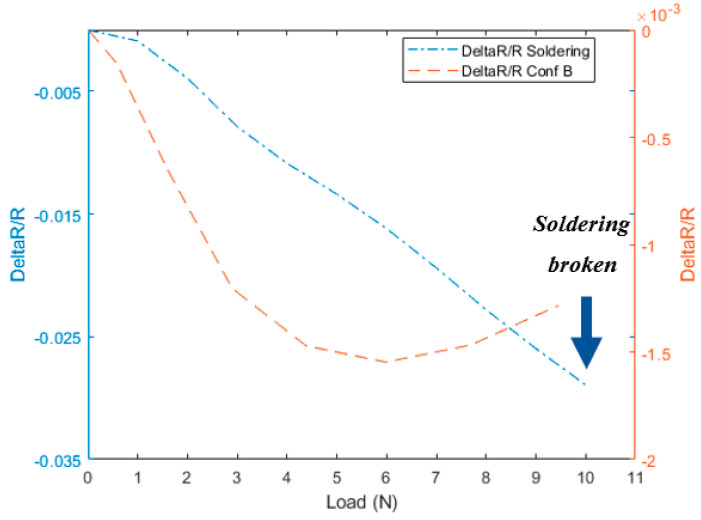
The tensile tests under soldering and configuration B.

The “free CF length” ends by a high jump in the resistance due to the sudden fracture of the fiber called the “breakage” region. The disparity between the three configurations in Figure 4, Figure 5 and Figure 6 comes through the determination of the minimum strain needed to start each region for the different configurations. For instance, the two configurations A and C show a relative delay in starting the “free CF length” region i.e., Figure 4 and Figure 6, compared to B configuration in Figure 5. This is due to the partial gripping in A and non-uniform gripping in C configuration caused by the whole coverage of aluminum. While in B configuration, the aluminum covers only the CF under uniform tightness. The “free CF length” can be considered as the operating region for the CF where the resistance measurements are increasing and clearly reflecting the behavior of the CF under the tension load. For this reason, configuration B has been adopted for the next investigations in this study. We have also investigated measurements using direct gripping e.g., soldering the carbon fiber with the copper electrode using C-solder lead, shown in Figure 2A. These measurements have been limited to a small value of load i.e., 10 N as a result of the systematic sudden breakage of the soldering. Therefore, the load was applied in increments of 1 N. In contrast, the load was continuously applied for samples prepared using configuration B in Figure 3. Accordingly, the results for resistance variation vs load are presented in Figure 7 for C-soldering and configuration B gripping system, respectively. There is a noticeable difference in the resistance variation between the two gripping systems. The variation of the resistance under the same loading increment is much lower in Plexiglas gripping (configuration B) compared to direct C-soldering. This complements the justification of the use of configuration B discussed previously.

### 3.2. Electromechanical Characterization of the Carbon Fiber

**Figure 8:**
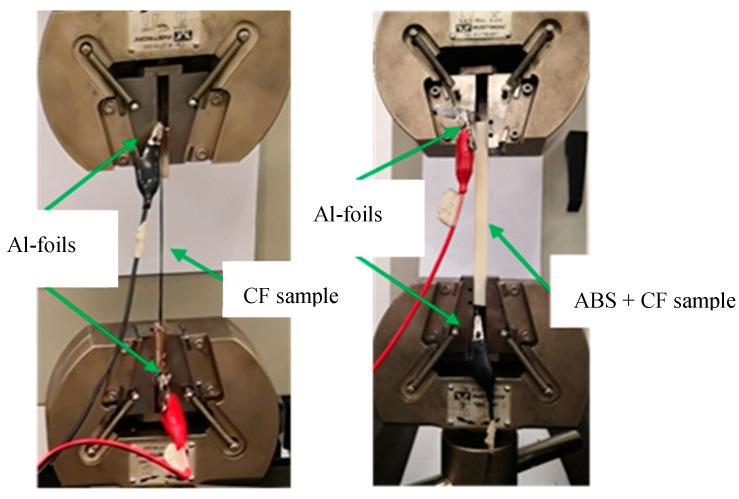
Tensile test setup.

The tests were conducted with simultaneous records of the load applied in tension to the bare fiber while measuring the electrical resistance variations for characterization. The typical setup of the tests is shown in Figure 8. Similar tests have been carried out with the embedded filament in the polymer e.g., ABS.

#### 3.2.1. Standalone Carbon Fiber Tow under Test

**Figure 9:**
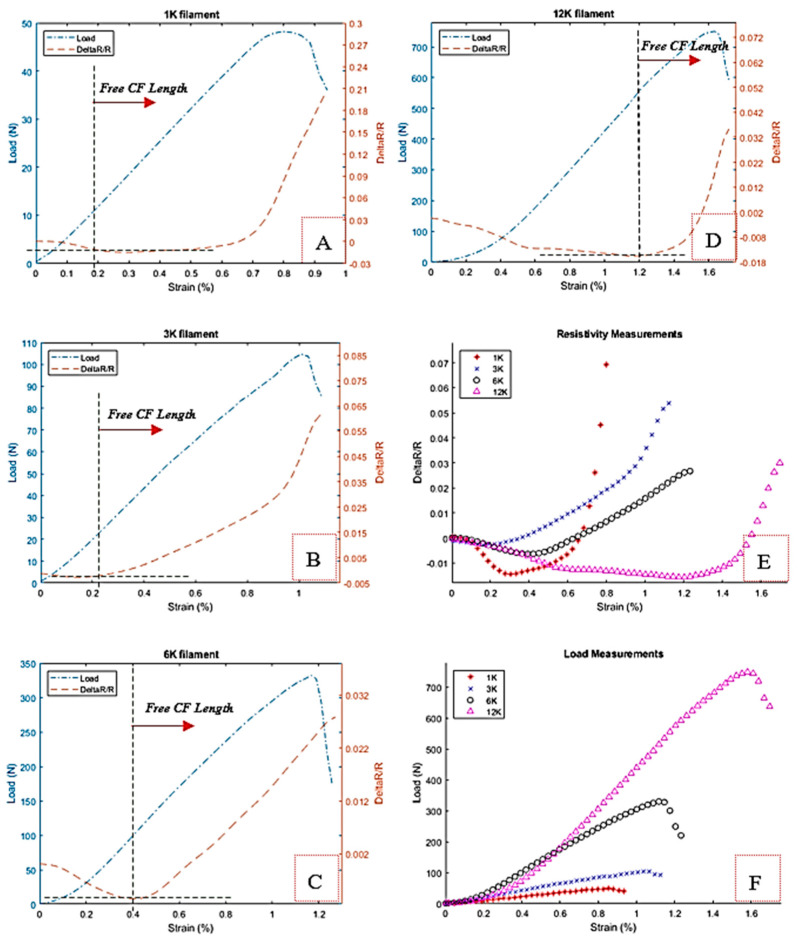
Bare fiber resistance change and the tensile test load versus the tensile strain for (**A**) 1 K, (**B**) 3 K, (**C**) 6 K, (**D**) 12 K, and combined results for all tow filaments versus strain; (**E**) resistances and (**F**) loads.

**Figure 10:**
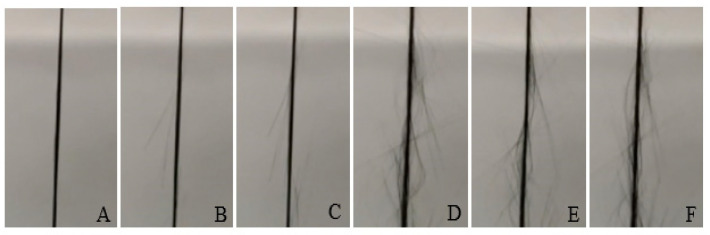
Pictures for the 1 K filament during the tensile test (**A**) before breakage, (**B**) partially broken, (**C**–**F**) successive pictures for the filament until complete breakage.

The four categories 1, 3, 6, and 12 K are considered in a standalone test. Figure 9A–D shows the behavior of the carbon fiber filament in various categories under the tensile test as well as the variation of the resistance with respect to strain. The resistance variation curves are not linear compared to the load curves due to the fact that breakage starts to appear gradually, as shown in Figure 10, on each filament and hence affecting more the resistance rather than load since we are showing the relative variation of the resistance that is more sensitive than the total load.

Based on the observation made previously in the preliminary results for the gripping, a cyclic tensile test has been conducted for the CF. The results are discussed later. The 1 K filament exhibits a leading strain to start almost immediately the free CF length region, followed by 3, 6, and 12 K, respectively, with different behaviors. Based on the idea that the through-thickness phenomena is closely related to the number of filaments in one tow, the number of fibers contributes significantly to that behavior, where the probability of the contacts among the fibers increases in the 1, 3, 6, and 12 K filaments, respectively.

The higher number of fiber filaments requires high strain to overcome the effect of the through-thickness. Figure 9E,F compares the resistance variation and load applied with respect to strain for all filament types. It is clear that the 12 K filaments exhibit higher strain and load for fracture that is reasonable due to its characteristic of possessing a higher volume of filaments material. The corresponding relative resistance goes through a clear negative ratio compared to others to move in the free length region to positive until the fiber breaks. With this negative relative resistance variation R/R at the gripping area, the carbon fiber acts as an auxetic material with negative Poisson ratio under the preload of the grips.

The response of the carbon fiber for the tensile load can be represented by the change of the electric resistance. Theoretically and based on Equations (1) and (2), these changes may happen due to either geometrical changes which are summarized by Poisson’s ratio and the strain, or material properties, precisely the resistivity change or piezo-resistivity. The opposite behavior of the resistance measurements at the beginning of the graph is being attributed to either the through-thickness resistance phenomena, which appears wherever the fibers are in contact, but this may not be the case since we apply a pretension force that can eliminate this cause or at least reduce its effects. In addition, the usual resin application is absent. No such observation was found in the literature since results with this type of fiber clamping were not published before. The cause may be due only to gripping forces. However, Wang et al. observed this auxetic behavior for CF in epoxy-matric composite under compression [5]. As it has been stated in preliminary tests, our gripping systems have a connectivity purpose in addition to the clamping. Based on that, the carbon fiber was divided into two main regions, see Figure 9A–D; the carbon fiber within the clamping region which we called the gripping region and the free-length region.

The first part is the critical region in the gripping. It was noticed that the variation of the resistance is almost negative due to possibly the compressive stress developed under the clamps as shown in Figure 9E. The compressive stress due to clamping contributes to this variation of the resistance. In this context, the rough surface between the fiber and the electrode allows possibly the release of the compressed cross-section of the fiber more than the possible length extension (pulling) that is still in the elastic domain and in limited change, hence the resistivity denoted by the ratio ρL/A decreases locally. This will continue until the clamps lock the releasing of the carbon fiber in the gripping region to be under full tension and to continue on the actual pulling in the free length region defined as the second part. This part will exhibit a normal behavior after a while of the test starting point where it will predominately contribute to the resistance change i.e., positive and almost linear slop region in the graphs, see Figure 9F.

The breakage progress of the carbon fiber under tensile test is shown in Figure 10. A couple of fibers start to break, affecting both electrical resistance and pulling load. In Figure 10F, the fiber is about to fail completely. This phenomenon happens usually around the middle of the sample.

#### 3.2.2. Fiber Embedded in ABS Host Material

**Figure 11:**
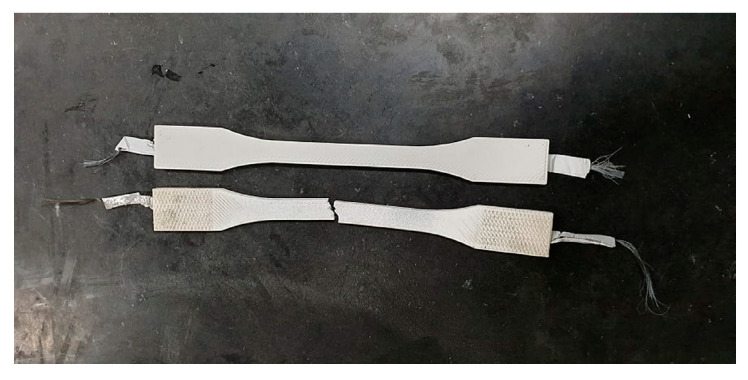
ABS sample with embedded carbon fiber as a sensor.

In the second phase of the work, the aim is to investigate the fiber embedded inside a host material, the tensile tests have been carried out on the ABS samples. Standard samples were prepared with the host ABS polymer (Table A2) using a commercial 3D printer. A dog bone shape sample for uniaxial tension test under ISO 527-4; 1997 has been built and shown in Figure 11. The figure shows also a broken one after test. As it can be seen, a thin aluminum sheet is added at the interface between the fiber and ABS towards the attachment area and connection ports similar to configuration B in Figure 3. Table 1 summarizes the specifications of the samples used. The relative densities for the samples are within the range of 92.5 ± 0.7%. The embedding of CF does not contribute much to the axial strengthening of the ABS samples when they are prepared with 100% filling during printing, see Table 1. The tensile strength improves by almost 4%. The strengthening effect appears well only when the filling percentage is low e.g., 20–50% where strength will be governed by almost the carbon fiber, or in bending. The initial electric resistance was measured for each embedded fiber before running the tensile test; the last column in Table 1 clearly exhibits the effect of the embedding on the initial resistance before the test compared with the bare fiber resistance.

**Figure 12:**
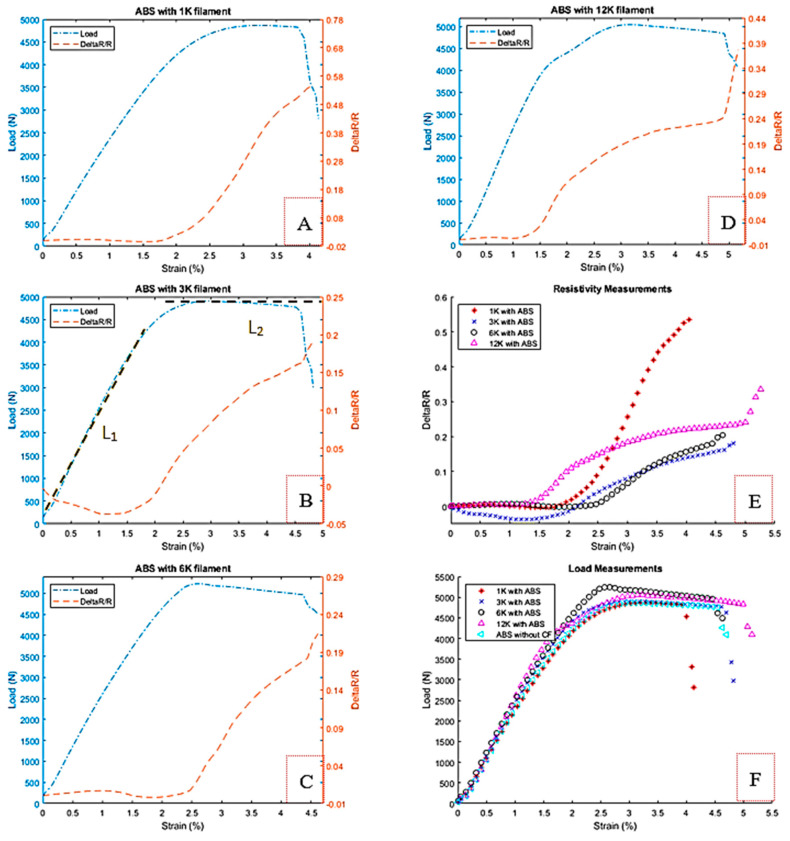
Resistance change and the tensile test load verses the tensile strain for ABS embedded with (**A**) 1 K, (**B**) 3 K, (**C**) 6 K, (**D**) 12 K, (**E**,**F**) Combined results for all filaments.

Figure 12 shows the behavior of the different types of filaments under the tensile test and resistance measurement. The tensile test shows almost a linear region of the load with respect to the strain, arriving to the yielding region and breaking after that from the second linear behavior as shown in Figure 12B lines L_1_ and L_2_. The relative resistance is not straightforward due to the gripping inside the polymer at the clamping area. The relative resistance starts with a constant towards a negative slope and then positive as seen previously with standalone tow carbon fibers. Originally, the 1 K tow filament and due to its relatively small size i.e., less contact between the fibers, are not much affected by the through-thickness phenomena and also the embedding process, where the negativity of the relative resistance is very low. The previous results for the bare fiber filaments show that increasing filament’s number leads to an increase in the effect of the through-thickness. Accordingly, the negative ratio for resistance variation will appear; for instance, in the 3 K filament tow shown in Figure 12B. The embedding process reduces these effects for the 6 K and 12 K filaments, where the good embedding needs a spreading the fibers for such sizes of filaments during the printing process which leads to decreasing the negativity compared with the 3 K filaments. On the other side, we have observed the gripping effect on the resistance measurements in the 1, 3, and 6 K filaments in Figure 12A–C, which is relatively high compared to Figure 12D. The relative resistance increases simultaneously with the strain in this particular case because of the high volume of the filaments in the 12 K compared to others. Figure 12E,F summarizes the difference in both strain and load for all ABS with embedded filaments.

### 3.3. Cyclic Tensile and Resistance Test

**Figure 13:**
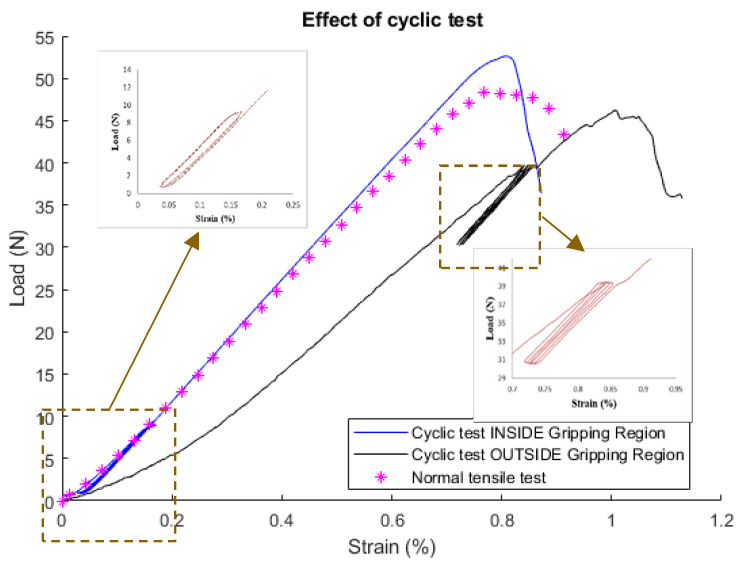
Cyclic test outside the gripping region with hysteresis behavior.

We investigated the load cycling effect on the gripping region and outside in the free length. The cyclic tests have been carried out within the load limits for those regions. The load between (0–10) N was applied for the inside gripping region in Figure 13, and for the range (30–40) N for outside the gripping region as shown in Figure 13. The cycling in both cases is repeatable conserving similar amplitudes.

However, if we compare the hysteretic behavior captured for both cases in Figure 13, considering the load and the relative resistance, the inside gripping region displays almost an elastic behavior with very low hysteresis losses. As opposed to that, the outside free length CF shows a plastic behavior with the effect of hysteresis that can be seen in Figure 13.

**Figure 14:**
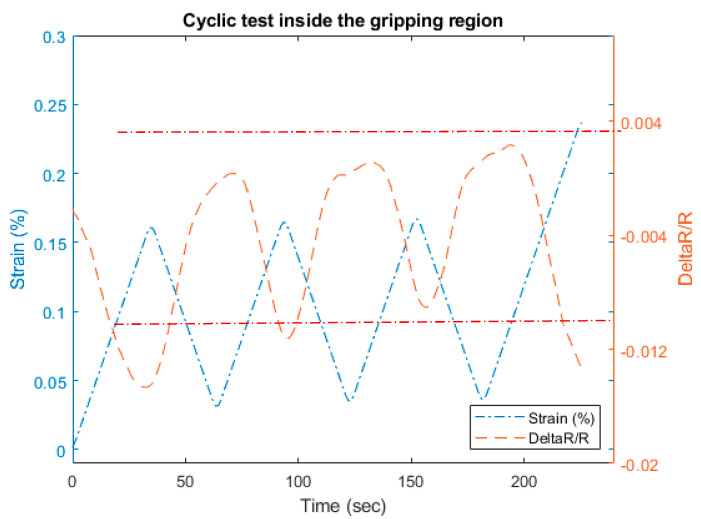
Resistance measurements for the cyclic test inside the gripping region.

**Figure 15:**
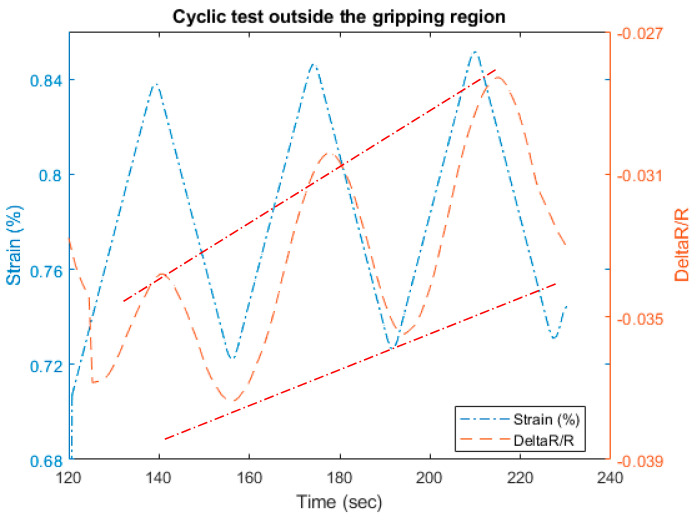
Resistance measurements for the cyclic test outside the gripping region.

This characteristic can be used to monitor structural damage of the host material if the CF is embedded. The negative behavior of the resistance during the inside region has been approved again in Figure 14; where the applied tensile strain leads to unusual resistance changes i.e., the increase in strain leads to a decrease in resistance measurements and vice versa. On the other hand, the resistance shows a good compatibility to the applied strain in the outside gripping region, shown in Figure 15. The resistance does not return to its original value due to some irreversible damages in the fibers [40].

## 4. Temperature Measurements under Tensile Test of the Carbon Fiber

**Figure 16:**
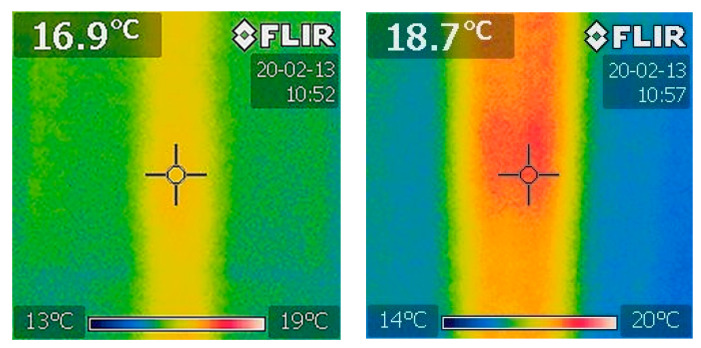
Temperature measurements of the carbon fiber under tensile test.

Since the carbon fiber material is subject to a pulling force under tensile test, Equation (2) shows the components of the relative variation of the resistance depending on the strain and resistivity. The variation of the resistivity can also be governed by the effect of temperature as in Equation (3):

(3) $$\rho =\rho_{0}\left(1+\alpha \times \Delta T\right)$$

where α is the thermal expansion coefficient and ΔT the variation of the temperature where the material was subject to. This temperature is generated through the deformation of the carbon fibers. With this effect casted in this equation, we have measured then the variation of temperature on the fiber as the tensile test was progressing using a thermal FLIR camera located in the middle of the sample. We have considered 50 K type since it is a large density of filament and allows the thermal camera to measure its temperature as the tensile test progresses as shown in Figure 16. The previous types of 1, 3, 6, and 12 K were too thin to receive the infrared light reliably. Hence, they were not considered.

**Figure 17:**
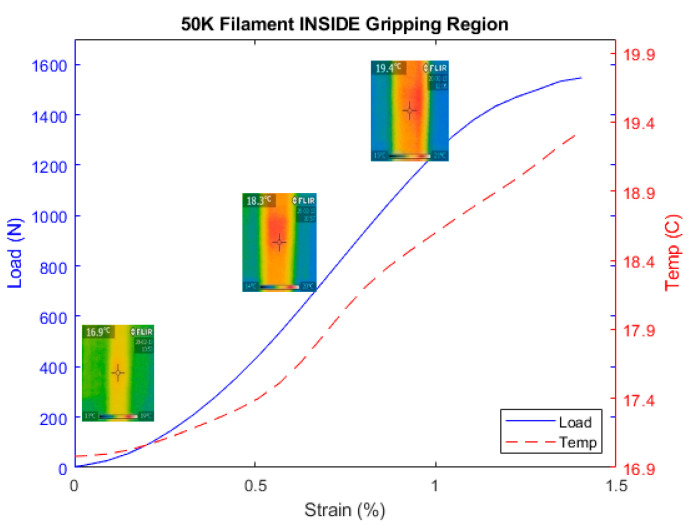
Load and temperature measurements of the 50 K carbon fiber under full tensile test.

**Figure 18:**
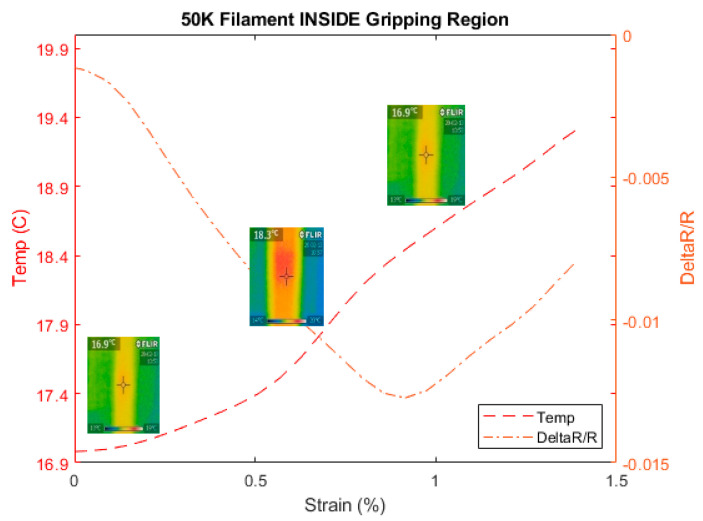
Temperature and relative resistance variation for the 50 K carbon fiber.

**Figure 19:**
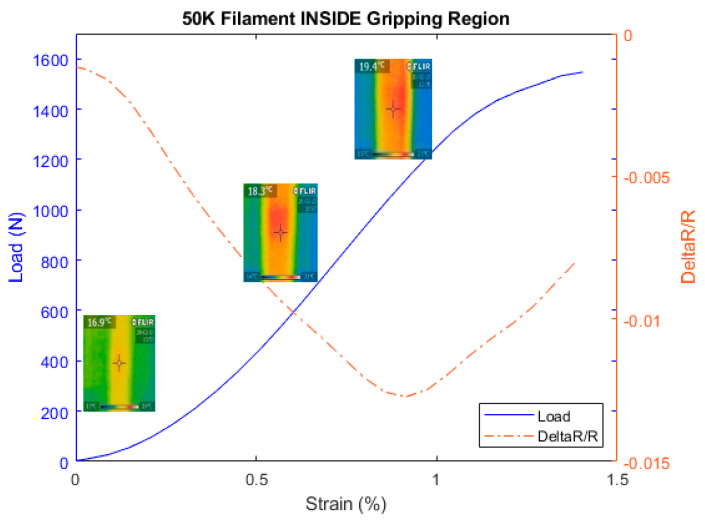
Load and relative resistance variation for the 50 K carbon fiber.

Figure 17 shows the temperature increase of the carbon fiber as the tensile test progresses to confirm Equation (3). Simultaneously, the electrical resistance was measured for the carbon fiber, as shown in Figure 18 to investigate the effect of the temperature on its resistance during the tensile test with special focus at the gripping region. Figure 19 confirms the observations made previously regarding the use of the configuration B for 50 K carbon fiber. Based on Equations (2) and (3), the main components affecting the behavior of the carbon fiber during tensile test are the Poisson ratio with major contribution and the piezo-resistivity that also depends on the temperature based on Equation (3). In addition, regarding the minimum value of strain needed to overcome the negativity in the gripping region is about 0.8% that is relatively high compared to the other types of CF filaments with the reason to be due to the through-thickness phenomenon mentioned before where the probability of the contact between the fibers is higher.

Figure 19 shows the behavior of the electrical resistance decreasing and increasing inside the gripping region at the cutoff of free CF length at 0.8%, while the temperature is rising as discussed in the previous tests. This rise of the temperature is triggered partly by CF under tight condition inside the gripping region and subject to both pulling and the extension of the material itself. It is observed that the cutoff has expectedly increased with 50 K tow to 0.8% compared to the previous announced value of strains of 0.18%, 0.23%, and 0.4% required for 1, 3, and 6 K filaments, respectively.

On the temperature contribution to the electromechanical characteristics, it is evident that there is a variation in the temperature during the tensile test. Although the variation is negligible for low number of filaments, it is noticeable in large tows density. This increase of temperature may affect the precision of measurements in applications e.g., damages in cement or concrete structures or when subject to harsh environment. Further analysis is needed to explore this effect. Currently, CFs are embedded manually into host material during 3D printing. There is a need of a well-defined process of embedding where the embedded fibers are fed in the host material with prescribed initial tension. For the sensing precision measurement, further investigations are needed to check the effects of this generated temperature with respect to the host material temperature on the electrical resistance measurements.

## 5. Conclusions

This paper investigated the electromechanical characteristics with measurement of an induced temperature variation during the tensile test of non-pretreated bare carbon fibers and embedded in host ABS material using 3D printing. A particular emphasis on the clamping of the carbon fiber for the tensile tests has been taken into consideration since this has been discussed in the literature and we face it in the current tests.

Careful attention has to be considered when designing the clamping of the ports for reading to avoid any secondary issue that may affect free length measurement. With this type of clamping selected, the carbon fiber has exhibited two regions with different behaviors; one related to the clamping with negative electrical resistance variation and positive loading since it is pre-loaded. The second one was related to the free effective length of the fiber. To overcome this gripping region, a minimum value of strain should be applied. This value depends on the number of fiber filaments; for instance, a strain of 0.18%, 0.23%, and 0.4% was required for 1, 3, and 6 K filaments, respectively. Similar behavior has been noticed when CF was embedded in ABS polymer. Abnormally, the 12 K filaments exhibited a difference for both bare CF or ABS embedded CF. A relatively minimum high strain of 1.3% is needed to overcome the gripping region in bare CF.

The negative behavior of the resistance has affected the cycling tests where an opposite behavior of deformation to the relative resistance variation appears inside the gripping region. However, good agreement for the actual behavior of deformation was depicted outside the gripping region. Linear behavior was observed for CFs embedded inside ABS material in that region. Adherence of the ABS to CF is key. This might be promising since it will allow the embedding of fibers without the need of pretreatment process, hence, reducing fabrication time and cost. 

Several more composites that have been investigated, e.g., polyactide PLA, have shown that with a secured adherence at the interface with the CF and showing sensitive tensometric properties, little shifted from ABS in tensile and bending are suitable for measurement. The group of composites includes fiber-reinforced composites e.g., ABS, polycarbonate (PC) and polyphenylsulfone (PPSU), as well as the epoxy resin composites. 

The CF seemed to behave like an auxetic material with negative Poisson ratio under the preload of the grips. This indicates the need for proper fixing mechanisms for the fibers when embedded in polymeric materials. Moreover, initial pre-tension is essential to avoid the initial gripping region and allow the fibers to operate in the linear range with improved sensitivity. However, there is a need to examine the effectiveness of fibers under cyclic load since breakage of fibers is inevitable.

## Figures and Tables

**Table 1:** ABS samples’ specifications.

Samples	Relative Density (%)	Tensile Strength (MPa)	Initial Resistance (Ω)	Bare CF Resistance (Ω)	% difference between Initial and after Embedding
100% without fiber	91.4	25.27	-	-	-
100% with 1 K	92.1	25.6	48.59	44.17	10
100% with 3 K	92.5	26.03	16.98	14.72	15.33
100% with 6 K	92.7	26.14	8.09	7.36	9.91
100% with 12 K	93.3	26.35	4.33	3.68	17.66

**Figure A1:**
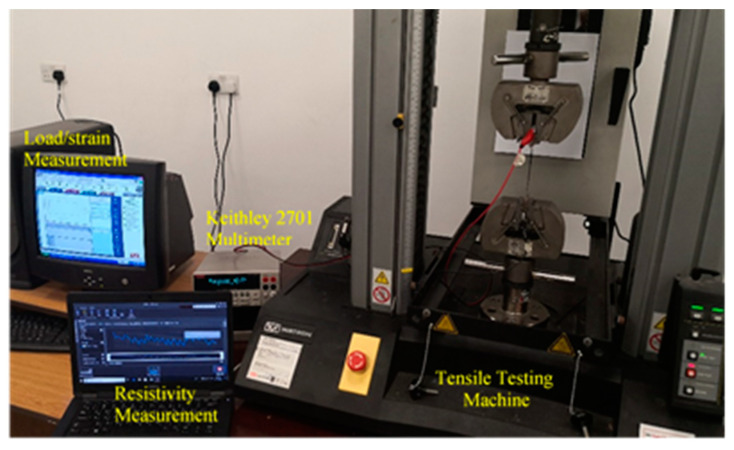
Experimental setup.

## Appendix A

### Experimental Setup and Material

The samples were prepared for tensile tests using INSTRON 5569-J6883 test machine. In parallel to that, the change of the CF resistance was planned to be measured and recorded during the tensile test using the multi-channel Keithley 2701 digital multimeter, see Figure A1.

For this investigation, the carbon fiber selected from off-the-shelf products is a polyacrylonitrile (PAN)-based commercial carbon fibers filaments. These filaments were in categories of 1, 3, 6, and 12 K related to the number of fibers per category. Each carbon fiber filament has a diameter in the range of 5 to 8 µm. They are identical in terms of their properties as shown in Table A1, but these properties will differ per category. The tests have been planned to be in two cases; direct test of CF without any prior treatment, and embedded CF inside a thermos plastic material; acrylonitrile butadiene styrene (ABS) using 3D printing. The properties of the ABS host material are shown in Table A2.

**Table A1:** General properties of the fiber filaments.

Filaments	Tensile Strength (MPa)	Tensile Modulus (GPa)	Tensile Strain (%)	Filament Diameter (µm)	Density (g/cm^3^)	Electric Conductivity (Ω.m)
1 K, 3 K, 6 K, 12 K	3530	230	1.5	7	1.76	1.7 × 10^−5^

**Table A2:** ABS material properties.

Yield Strength (Pa)	Tensile Strength (Pa)	Tensile Strain (%)	Young’s Modulus (Pa)	Density (g/cm^3^)	Print Temp. (°C)	Bed Temp. (°C)
1.85–5.10 × 10^7^	2.76–5.5 × 10^7^	5.5–6	1.9–2.9 × 10^9^	1.01	230–250	110
